# Impact of a community pharmacist-led medication review on medicines use in patients on polypharmacy - a prospective randomised controlled trial

**DOI:** 10.1186/s12913-016-1384-8

**Published:** 2016-04-23

**Authors:** Markus Messerli, Eva Blozik, Noortje Vriends, Kurt E. Hersberger

**Affiliations:** Pharmaceutical Care Research Group, Department of Pharmaceutical Sciences, University of Basel, Basel, Switzerland; Division of General Practice, Department of Medicine, University Medical Centre Freiburg, Freiburg, Germany; Division of Clinical Psychology and Psychiatry, Department of Psychology, University of Basel, Basel, Switzerland

**Keywords:** Polypharmacy, Community pharmacy, Medication review, Drug-related problems, Adherence to medication, Medicines use, Pharmaceutical care

## Abstract

**Background:**

In 2010 the ‘Polymedication Check’ (PMC), a pharmacist-led medication review, was newly introduced to be delivered independently from the prescriber and reimbursed by the Swiss health insurances. This study aimed at evaluating the impact of this new cognitive service focusing on medicines use and patients’ adherence in everyday life.

**Methods:**

This randomised controlled trial was conducted in 54 Swiss community pharmacies. Eligible patients used ≥4 prescribed medicines over >3 months. The intervention group received a PMC at study start (T-0) and after 28 weeks (T-28) while the control group received only a PMC at T-28.

Primary outcome measure was change in patients’ objective adherence, calculated as Medication Possession Ratio (MPR) and Daily Polypharmacy Possession Ratio (DPPR), using refill data from the pharmacies and patient information of dosing.

Subjective adherence was assessed as secondary outcome by self-report questionnaires (at T-0 and T-28) and telephone interviews (at T-2 and T-16), where participants estimated their overall adherence on a scale from 0–100 %.

**Results and discussion:**

A total of 450 patients were randomly allocated to intervention (*N* = 218, 48.4 %) and control group (*N* = 232, 51.6 %). Dropout rate was fairly low and comparable for both groups (*N*_Int_ = 37 (17.0 %), N_Cont_ = 41 (17.7 %), *p* = 0.845). Main addressed drug-related problem (DRP) during PMC at T-0 was insufficient adherence to at least one medicine (*N* = 69, 26.7 %). At T-28, 1020 chronic therapies fulfilled inclusion criteria for MPR calculation, representing 293 of 372 patients (78.8 %). Mean MPR and adherence to polypharmacy (DPPR) for both groups were equally high (MPR_Int_ = 88.3, SD = 19.03; MPR_Cont_ = 87.5, SD = 20.75 (*p* = 0.811) and DPPR_Int_ = 88.0, SD = 13.31; DPPR_Cont_ = 87.5, SD = 20.75 (*p* = 0.906), respectively).

Mean absolute change of subjective adherence between T-0 and T-2 was +1.03 % in the intervention and −0.41 % in the control group (*p* = 0.058). The number of patients reporting a change of their adherence of more than ±5 points on a scale 0–100 % between T-0 and T-2 was significantly higher in the intervention group (N_Improvement_ = 30; N_Worsening_ = 14) than in the control group (N_Improvement_ = 20; N_Worsening_ = 24; *p* = 0.028).

**Conclusion:**

Through the PMC pharmacist were able to identify a significant number of DRPs. Participants showed high baseline objective adherence of 87.5 %, providing little potential for improvement. Hence, no significant increase of objective adherence was observed. However, regarding changes in subjective adherence of more than ±5 % the PMC showed a positive effect.

**Trial registration:**

Clinical trial registry database, NCT01739816; first entry on November 27, 2012.

**Electronic supplementary material:**

The online version of this article (doi:10.1186/s12913-016-1384-8) contains supplementary material, which is available to authorized users.

## Background

Increasing complexity of both, the therapy (polypharmacy) and the patient (multimorbidity) raises the risk for drug-related problems with adverse events and medication errors [1, 2]. Avoidable problems usually do not result from individual misconduct, but from suboptimal processes. Drug-related morbidity as a result of these risks is associated with high healthcare costs [3–5]. Situations with a high risk for  drug-related problems (DRP) include polypharmacy, significant changes in drug therapy or changes in existing diseases, insufficient response to drug therapy, suspected lack of therapy, symptoms of side effects, as well as discharge from hospital with a change of drug therapy [6, 7]. One approach to reduce the risks for developing DRP is to conduct medication reviews [8–10]. A worldwide shift in the professional role of pharmacists is observed [11]. Pharmacists participate increasingly in clinical processes and perform tasks in patient care. This transformation of the profession includes co-responsibility in the achievement of therapeutic success, cost efficiency and avoidance of drug-induced (re)hospitalisation. Accordingly, the Pharmaceutical Care Network Europe (PCNE) felt the need to redefine pharmaceutical care as “the pharmacist's contribution to the care of individuals in order to optimise medicines use and improve health outcomes” [12]. In the early 1990s, pharmaceutical care was introduced in community pharmacy practice in Switzerland. Emphasis was given to providing patient-centred care and cognitive services [13]. A postgraduate education program and mandatory continuous education were launched together with changes to pharmacists’ remuneration, which link payments to services delivered and not only to the volumes of medicines dispensed. In 2010, the current remuneration system was introduced, which defines a fee schedule for a total of nine distinct services. Among these services the so called ‘Polymedication Check’ (PMC) was newly introduced as the first cognitive service to be delivered by pharmacists independently of the prescriber for patients on ≥ 4 prescribed drugs taken over ≥ 3 months. In addition, the pharmacist may suggest - among other interventions - to provide the medicines in a weekly dosing aid (WDA) refilled by the pharmacy. Both services, the PMC and the weekly filling of a dosing aid by the pharmacist are reimbursed by the health insurance in the basic insurance. Moreover, the current regulation allows repeated dispensing of prescribed medicines for a maximum of 12 months. Currently, such prescriptions constitute nearly 75 % of all items dispensed [14]. Hence, Swiss community pharmacies assume very responsible roles in the care of chronic patients.

### Adherence and consequences of non-adherence

Approximately 25 % of patients with different diseases do not take their medication as prescribed, although the extent varies between 0–95 % [15]. On average, adherence in long-term therapy is 50 % [16]. Lack of adherence is the most common cause of the efficacy-effectiveness gap [17], defined as the gap between therapy efficacy in daily life compared to the effectiveness shown in clinical trials. Previous studies have shown a positive impact of structured interventions to improve adherence provided by pharmacists [18, 19]. But there is still little evidence related to the effectiveness of interventions performed in community pharmacies. A recent Cochrane review revealed that only a minority of studies with lowest risk of bias (RCT design) improved both adherence and clinical outcomes [20]. However, adherence as an outcome remains challenging to measure because of methodological issues and multifactorial influences [21]. Support of adherence to treatment is only successful if the entire medication is taken into account. Therefore, conducting a medication review is the essential first step in any adherence counseling.

### Medication review

According to the current PCNE definition, a medication review is ‘an evaluation of a patient‘s medicines with the aim of optimising medicines use and improving health outcomes. This entails detecting drug-related problems and recommending interventions.’ [22]. The analysis in a medication review always includes an inventory of current medicines, a history of complaints, their course, a patient’s concerns and individual needs for support. With respect to the pharmaceutical care process [12, 23], the medication review is the starting point leading to the suggestion of solutions, the planning and implementation of interventions and ultimately to the evaluation of the outcomes [24]. Pharmacist-led medication review services are available in several countries such as the United Kingdom (Medicines Use Review, MUR) [25], United States of America (Medication Therapy Management, MTM) [26, 27], Australia (Home Medication Review, HMR) [28], Canada (MedsCheck) [29, 30], and New Zealand (Medicines Use Review, MUR) [31]. According to a recent meta-analysis, a majority (57.9 %) of fee-for-service pharmacist-led medication reviews improved medication adherence and positively influenced patient outcomes [24].

### The Polymedication Check

The Swiss Polymedication Check (PMC) is based on the well-established Medicines Use Review (MUR) from United Kingdom [32, 33]. Information is available from the medication history, which is mandatorily kept in community pharmacies and from a structured patient interview. The Swiss PMC focuses on adherence problems, patients’ knowledge and handling problems and is followed by specific interventions or recommendations by the pharmacist. Implementation of such cognitive services provided by a pharmacist is known to be very challenging [34–36]. The same is true for Swiss community pharmacies. Implementation of the PMC is low and after three years only about three checks per pharmacy per year were registered, with a large majority of pharmacies not offering this service. While in 2011 2’534 PMCs were carried out, in 2014 the number of PMCs provided amounted at 6'940 PMCs [37], which is an encouraging trend.

### Rationale for the study

In Switzerland, new services remunerated by the basic health insurance require a proof of their efficacy, appropriateness, and economic effectiveness according national criteria [38]. The present study aimed at investigating the impact of the PMC on patients on polypharmacy. It was hypothesised that PMC would increase objective and subjective adherence in a community sample.

## Methods

### Trial design

A prospective, parallel group randomised controlled trial (RCT) design was chosen to evaluate the impact of the PMC. Contemporaneously, an in-depth evaluation of the process and the perspectives of patients and pharmacists was planned to collect information for further development of the service. The study setting considered community pharmacies in a range of representative regions of Switzerland (with and without self-dispensing physicians, city versus country, German-speaking part (D-CH) versus French-speaking part of Switzerland (F-CH)). For each patient the observation period lasted 28 weeks from study start (T-0) until study end (T-28).

### Eligibility for study pharmacists

The recruitment of 70 pharmacists was intended; thus, community pharmacies in the cantons Aargau (AG), Basel-Land (BL), Basel-Stadt (BS), Solothurn (SO), Fribourg (FR), Neuchâtel (NE), Genève (GE), Vaud (VD) und Valais (VS) were invited to participate in the study. Basing on the principle of "first in, first served", the ideal recruiting target was 50 pharmacists from the German speaking and 20 from the French speaking part of Switzerland in line with the national proportion of the population. Study pharmacists were required to take part in a study-specific training, and to give written consent regarding the study design as well as a memorandum of understanding through the pharmacy owner to collaborate on the project until the end of study; in addition, they were asked to commit to transfer patient’s refill data to the study centre, and to collaborate with either IFAK or OFAC (the two main clearing companies in Switzerland administering the charges between pharmacies and health insurance and therefore also holding the corresponding patient data). The three-hour training session provided by the study centre included an overview over the study, highlighted the need for compliance to the study protocol, and clarified rights and responsibilities of the study pharmacists. No further training on the execution of a PMC was offered as the study aimed at assessing and evaluating current practice.

### Screening for eligible patients

**Fig. 1:**
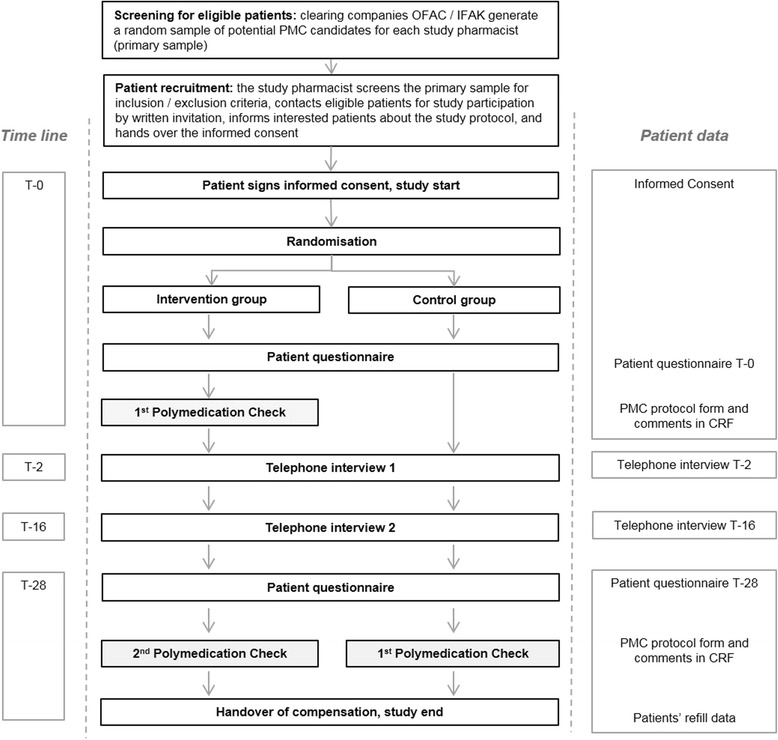
Study flow chart with screening and recruitment process

In order to avoid selection bias through study pharmacist (e.g. Individual prejudices, preferences), a random sample of 100 potential PMC candidates (age >18, ≥4 prescribed drugs for ≥3 months) was created for each study pharmacy in collaboration with the two main clearing companies IFAK and OFAC. The latter performed an independent screening for each study pharmacy and listed all patients fulfilling the selection criteria for a PMC. Out of this sample of potential PMC candidates, a random primary sample of 100 was selected by IFAK and OFAC (Fig. 1).

### Patient recruitment

The study pharmacist checked this primary sample for exclusion criteria and consecutively invited subsamples of ten patients by a letter to participate in the study. Exclusion criteria for final recruitment were the following: living in a retirement home, prior PMC, receiving weekly dosing aids filled by the pharmacy or another person, cognitive impairment, move or death, insufficient knowledge of written and spoken German or French. In addition, study pharmacists re-checked if a patient met the primary inclusion criteria. The study centre received information on gender, date of birth and the reasons for exclusion of a patient. If the patient had expressed his interest in the participation, the study pharmacist informed him about the schedule, potential risks, and compensation and handed over the declaration of consent.

### Randomisation process

The patients were assigned by 2 x 4 block randomisation into intervention or control group. Initially, each study pharmacist received two blocks containing eight dossiers (four intervention and four control) each packed in sealed and unlabelled envelopes. Once the first patient had consented, the study pharmacist opened one envelope out of the first block to reveal what arm of the study the patient had been randomised to. Once all eight envelopes of block No. 1 had been assigned, the next block was used. Upon request, further blocks were available.

### Structure of the intervention vs usual care

The intervention at T-0 included the execution of a PMC according to the official guidelines. The adapted study PMC protocol was used as assessment form. In a structured face-to-face counselling with the patient, the study pharmacists screened all medicines currently used. The pharmacists checked for any gaps in knowledge or other pharmaceutical care issues including handling and adherence problems. The interview took place in a separated area. Pharmacists were instructed to use open questions to detect pharmaceutical care issues and to decide if there was need for further investigation. For each medication, the PMC protocol (Additional file 1) required documentation whether the patient knew the reason why he/she took the medicines (yes/no), if he/she needed any counselling (yes/no) or had adherence problems (yes/no). Additionally, handling difficulties were enquired, and the pharmacist documented all resulting interventions such as consultation with the general practitioner (GP), referral of the patient, potential suggestion and implementation of a weekly dose reminder system, or any other recommendations or interventions. Where necessary, an individual patient education and a medication plan could be provided on the basis of the information gained from the interview. None of this follow-up interventions was standardised.

Usual care included no specific intervention and no documentation at T-0. Patients of the control group only received the two self-report questionnaires at study start and study end, and the two telephone interviews. Normal counseling for any new prescription or arising question from the patient was always allowed and guaranteed, so patients from this arm were not restricted from contacting the pharmacist for advice if they wished to do so. If a PMC became indispensable during the study period (e.g. by another pharmacist than the study pharmacist), this patient of the control group was excluded. Overall, the study took seven months for each patient and included two visits at the pharmacy with the completion of questionnaires and participation in two telephone interviews. Patients were able to contact the study centre in case of further interest for the study purposes or any problem with the study process (e.g. missed telephone interview) using a separate telephone hotline available 24 h seven days a week.

### Classification of detected drug-related problems and addressed interventions

To classify the addressed drug-related problems and describe the pharmacists’ interventions, the GSASA classification tool was used [39] This instrument comprises five main categories: i) problem, ii) type of problem, iii) cause, iv) intervention, and v) outcome. We adapted the category ‘causes’ by dividing the section ‘Insufficient knowledge of the patient’ into three subdomains focussing on patients’ individual needs for information about a) safe and effective use of his medicines b) the medicines’ potential adverse drug reactions c) his lifestyle, nutrition or empowerment in general. Further on, we added the category ‘More cost-effective therapy available’ as the recommendation of generic drugs might be likely triggered throughout a PMC.

### Case report forms for study pharmacists

In order to support study pharmacists in their compliance to the study protocol and to ensure coherent data capture, case report forms (CRF) were developed. The study pharmacist documented his interventions or recommendations resulting from PMC, classified the underlying problems according to their urgency (low, medium, high urgency) added any abnormalities or changes in the care of the patient.

### PMC protocol form

We used the official documentation form for PMC with minor changes to ease data capture for the purpose of the study (Additional file 1). This assessment form still showed the format of one A4 side. At study end (T-28), in addition to the PMC protocol the study pharmacist documented observed drug-related problems, the frequency of falls, and all changes in therapies since T-0 reported by the patient (dosage change, generic substitution, start/stop, no change). The documentation of these changes was needed to identify eligible therapies for objective adherence calculation.

### Patient self-report questionnaires

Patient self-report questionnaires were developed to collect demographic data (age, gender, living situation, education and employment status, smoking status), but also to describe his limitations in executing everyday activities (four items extracted form of the Disabilities of the Arm, Shoulder and Hand (DASH) questionnaire [40]) and assess his subjective adherence at T-0 and T-28. The patient therefore had to assess his adherence to all his prescribed medicines for the last two weeks using a visual analogue scale (VAS_AD_) 0–100 mm representing 0 for ‘taken none’ and 100 for ‘taken all my medicines’. Patients were asked to fill in the questionnaires in the pharmacy at T-0 and T-28, seal them in an envelope and return the envelope to the study pharmacist. Thus, the study pharmacists had no knowledge of the responses given by their patients.

### Telephone interviews

In collaboration with a clinical psychologist and an economist, two comprehensive in-depth patient telephone interviews were developed aiming at monitoring possible impact of the intervention on patient’s knowledge and medicines use. After literature research, the Rob Horne’s ‘Beliefs about Medicines Questionnaire’ [41] and two questions out of the ‘8-item Morisky Medication Adherence Scale’ (German version, 8-MMAS-D) [42] were defined as suitable to be used as validated questionnaires fulfilling our criteria for telephone interview 1. In addition, we developed new rating questions to report their adherence to their therapy management. Patient had to answer the same question as in the patient questionnaire T-0 to describe their adherence, but in a spoken percentage value. We also chose consistently a 10-item Likert scale. Options ranged from 1 (= ‘not at all’) to 10 (= ‘very much’). The response category ‘no answer’ was always available. Number of open questions (*N* = 7) was limited to ease documentation.

The first telephone interview contained 58 questions, divided into five sections: i) knowledge of their medicines and daily use, ii) subjective adherence estimation/use of reminder devices, iii) visits at general practitioner/hospital, iv) beliefs about medicines questionnaire, v) support by pharmacists. The interview 2 contained 53 questions, divided into the same sections as in the first interview. Compared with the first interview, 18 questions were excluded and 13 new questions were added. The telephone interviews were carried out two (T-2) and 16 weeks (T-16) after study start by clinical psychologists. The interviewers were blinded to the intervention and without any knowledge of the content of the PMC or the patient’s questionnaire T-0. A telephone interviewer’s coaching and monitoring of compliance with the study protocol was continuously provided by an independent academic psychologist as external expert. A structured interview guide was created using the software program Flexiform 2.6.9 to enable data entry during the interview. Piloting of all study documents and preparation of telephone interviews (recruiting interviewers, briefing and test interviews) were carried out in collaboration with the department of psychology of the University of Basel. All survey instruments were translated into French and retranslated into German to check for differences.

### Objective adherence measurement

Objective adherence rates based on refill data of the pharmacies and patient reported dosing regimen. Two methods for objective adherence calculation were used: a) Medication Possession Ratio (MPR) [43], calculated by dividing the days’ supply of a medication dispensed by the number of days in the time interval of interest, representing the adherence per each medicine and b) Daily Polypharmacy Possession Ratio (DPPR) [44], the proportion of time a patient had medication available for use by considering the presence or absence of multiple medications on each day in the observation period, representing the adherence per patient with his chronic polypharmacy. In this analysis only medicines were included, of which the patient reported at T-28 a daily use over the whole study period. Only oral drug forms with definite dosage where considered. Further, a prescription for the medicine had to be redeemed at least once before T-0. Therapies were excluded if prescribed by self-dispensing physicians (cantons BL/SO), changed in dosage during study period, chronic ‘on demand therapies’ (namely pain killers (ATC N02 and M01A), anxiolytics (ATC N05BA), or magnesium supplements (ATC A12CC). Also creams or drops where excluded from analysis due to imprecise assumption concerning dosing regimen. According to the theoretical calculation for both, the MPR and the DPPR, refill data was exported from the patient’s pharmacy. The export included the history of patient’s refills from at least 200 days before T-0 and the study period (T0 to T28, 196 days). For each dispensed medicine, the export comprised the date of refill, a product unique identifier number (pharmacode), the drugs’ ATC-Code, and the number of packages delivered. Subsequently, the pharmacode was matched with the Swiss index database GALDAT®/pharmINDEX® [45] to add the products’ package size (number of tablets) and complemented with the patient reported dosing regimen at T-28 (taken from the PMC protocol of both, intervention and control group). The calculation algorithm started with a look-back loop of 200 days before T-0 taking any packages of medicines postponed to the patient, equalising the fact that the patient was already on therapy before study start. As in previous trials, objective non-adherence was defined as MPR <80 % [46]. Also for the patient’s individualised aggregated measure DPPR, the cut-off for non-adherence was set <80 %.

### Subjective adherence measurement

Subjective non-adherence was defined in patient reported questionnaires (T-0 and T-28) as VAS_AD_ <100 m, in telephone interview 1 and 2 as Likert scale <10 and in telephone interview 2 additionally as 8-MMAS-D <6.00.

### Unplanned visits at the general practitioner/hospital

In order to evaluate a negative impact on the health system, patients’ unplanned visits at the general practitioner or hospital were assessed within the patient’ self-report at T-0 and T-28 and during telephone interview at T-2 and T-16.

### Sample size

To determine the required sample size, a power analysis was conducted. In the present study, the null hypothesis is rejected if the primary outcome adherence (as measured by MPR) improves by 5 % through the PMC on an assumed baseline MPR of 60 %. These suggestions were based on experiences from comparable projects [47]. We assumed a standard deviation of 20 % for both groups and used the conventional alpha error of 5 %. To have a statistical power of 80 % we would require 252 patients at T-28 in each group. Assuming a dropout rate of 35 % [48], this would lead to a total sample size of 780 at T-0 (calculated with http://sampsize.sourceforge.net). Thus, we expected from each study pharmacists an enrolment of 10–20 patients. There was no minimal/maximal number for recruited patients per study pharmacist.

### Statistical methods

Frequencies were evaluated using the chi-square test, ordinal scales were tested with the non-parametrical Mann-Whitney-*U*-test. The time course of the various endpoints was calculated using a general linear model (GLM) for repeated measurement method. The study groups were recorded as between-subject variable and the course of the corresponding values as within-subject variable in the model. In case of many missing values, individual templates mixed models analysis was chosen as an alternative method. All statistical tests were two-sided with a significance level of 5 %.

### Handling missing data

The intention-to-treat analysis included all enrolled subjects, divided into intervention and control groups. Patients were rated as a drop out when they were excluded at their request or when they were no longer available at study end. Reasons for drop out were documented if available. Patients who missed one or both telephone interviews remained in the study.

### Ethical approval

The study was approved by the responsible local ethic commission ‘Ethikkommission beider Basel (EKBB)’ (23.05.2012, registry number EKBB 50/12) as the leading committee for this multicentre study. Following the positive decision from the EKBB, the project was also approved by the local ethics committees of the following cantons: AG/SO (26.11.2012), VS (05.03.2013), VD/NE (12.03.2013), GE (22.03.2013), and FR (25.03.2013). The study was registered with the https://clinicaltrials.gov/ trials database (NCT 01739816). The fee for providing the PMC was covered by basic health care insurance. Study pharmacists received a compensation of CHF 150 for participating in the training session and CHF 50 for the delivery of each complete patient data set. Patients were paid CHF 20 for their time spent for the telephone interviews, and as a compensation for obligatory co-payment to the PMC-fee.

## Results

### Implementation of the study

Patient recruitment was conducted in three stages (BS, BL: July 2012 – February 2013, AG, SO: December 2012 – July 2013, the French speaking cantons (VS, VD, NE, GE, and FR): April 2013 – October 2013) and ended in April 2014 with the last patient completing the study protocol.

### Recruitment of study pharmacists and study pharmacies

Of 413 pharmacies invited for participation (N_BS/BL_ = 110; N_AG/SO_ = 135; N_VS/VD/NE/GE/FR_ = 168), 70 pharmacists signed the informed consent and were trained to follow the study protocol. In the end, 64 pharmacists (91.4 %) from 54 different pharmacies took part in the study (Table 1). Pharmacies were more or less evenly distributed between central (*N* = 15, 27.8 %), peripheral (*N* = 16, 29.6 %) and urban settings (*N* = 23, 42.6 %) as well as between being independent (*N* = 17, 31.5 %), belonging to a group (*N* = 23, 42.6 %), and belonging to a chain (*N* = 14, 25.9 %). A majority of 75 % of study pharmacists were women (*N* = 48), mean age was 42.8 years (SD 11.61), mean professional experiences working in a community pharmacy was 14.9 years (SD 10.69), and 27 pharmacists (42.2 %) had post graduate qualification in community pharmacy. The pharmacies showed variation in both size and infrastructure. Virtually all pharmacies were well equipped with a private area for the patients in terms of ensuring privacy from other patients (*N* = 51, 94.4 %). The median consulting area was 7 square meters (Range 1-25 m^2^).

**Table 1:** Demographics of study population at T-0, divided in language regions German-speaking (D-CH) and French-speaking (F-CH) part of Switzerland. The total sum per study group is highlighted in bold

	Intervention group (*N* = 218)						Control group (*N* = 232)						
	D-CH (*n* = 146)		F-CH (*n* = 72)		Sum		D-CH (*n* = 160)		F-CH (*n* = 72)		Sum		*p*Value
Women (n/%)	76	52.1	42	58.3	**118**	**54.1**	78	48.8	47	65.3	**125**	**53.9**	0.958
Living alone (n/%)	53	36.3	25	34.7	**78**	**36.5**	42	26.3	31	43.1	**73**	**31.9**	0.310
Smoker (n/%)	20	13.7	19	26.4	**39**	**18.5**	27	16.9	7	9.7	**34**	**15.0**	0.335
Age in years (Mean/SD)	66.4	11.38	68.7	11.73	**67.2**	**11.52**	67.1	10.80	67.2	13.18	**67.1**	**11.56**	0.845
Dash-4 score (Mean/SD)	4.7	1.72	5.3	2.43	**4.9**	**2.01**	4.7	1.48	5.3	2.40	**4.9**	**1.83**	0.323

### Patient recruitment

**Fig. 2:**
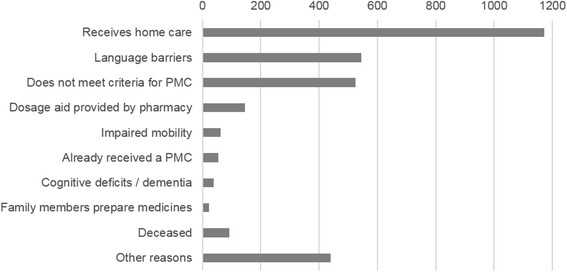
Pattern of reasons for exclusion after screening the random sample of potential candidates (*N* = 3096)

**Fig. 3:**
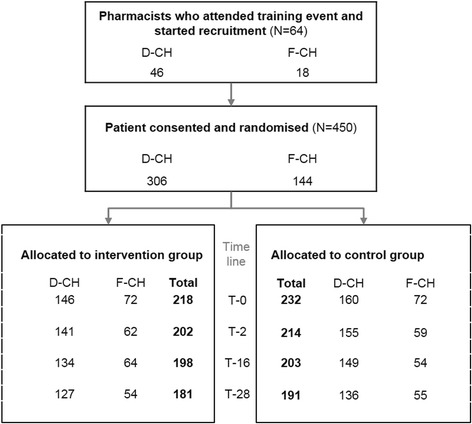
Recruited pharmacists and patients (D-CH = BS/BL/AG/SO, F-CH = GE/FR/NE/VD/VS)

For each pharmacy a random sample of potential candidates was delivered directly to the study pharmacist by IFAC and OFAC. The study pharmacists then consecutively checked samples of ten candidates for inclusion and exclusion criteria and invited the eligible patients. Exclusion criteria are available for 3096 patients as reported by 49 pharmacists (76.6 %) (Fig. 2). The other 15 pharmacists did not report about exclusions. After invitation, a total of 450 patients signed the IC and were randomly allocated to intervention (*N* = 218, 48.4 %) and control group (*N* = 232, 51.6 %) (Fig. 3). Median number of recruited patients per pharmacist was 7 (Range 1–17).

### Demographic data of recruited patients

Demographic data of recruited patients (*N* = 450) showed no significant differences between study groups (Table 1). The proportion of women living alone (111 of 243) compared to men (40 of 207) was significantly higher (*p* < 0.0001). Men showed a significantly lower DASH-4 score than women (Men_DASH-4_ = 4.5 (SD 2.09), Women_DASH-4_ = 5.2 (SD 1.60); *p* < 0.0001). No differences between groups observed concerning education and employment (data not shown).

### Dropouts

Out of 70 study pharmacists, six (8.6 %) withdrew before recruiting any patient for the study. While four stated that they had under-estimated the time amount to comply with the study protocol, two were no longer interested in the project. Dropout rate of patients was 17.3 % (*N* = 78); the different reasons for dropout are listed in table 2. Only 18 patients (4.0 %) withdrew from the study. The largest single cause for dropout of patients was that five of the 64 pharmacists who began recruiting quit the study (7.8 %), resulting in 17 patients lost in each group.

**Table 2:** Reasons for patient dropout summed at T-28, *N* = 78

	Intervention	Control	*p*Value
*Deceased*	2	2	
*Withdrawal by patient*			
- without information	5	7	
- lack of motivation/interest	1	1	
- poor health	0	4	
*Pharmacist was unable to collect data*			
- not achieved	5	5	
- patient has moved away	3	2	
- patient is in a nursing home	2	0	
- poor health	2	3	
*Lost because pharmacist revoke study participation*	17	17	
Total n (%)	37 (47.4)	41 (52.6)	0.845

### Intervention

Mean time per PMC was 29.8 min (SD 16.51; Range 5–135 Min). Mean number of chronic medication per patient was 6.8 (SD 2.92; Range 1–19), while 1.9 medicines (SD 2.07; Range 0–12) were prescribed on demand and 0.8 medicines (SD1.09; Range 0–5) were used as self-medication. A majority (*N* = 115, 52.8 %) revealed to be more time consuming than initial assumptions of the professional association, pharmaSuisse (>25 min). At T-0, study pharmacists reported 258 drug-related problems (1.18 per patient) they had discussed during the PMC. The two main causes of drug-related problems triggering counseling through study pharmacists were a) insufficient adherence to at least one medication of a patient’s polypharmacy (*N* = 69, 26.7 %) and b) lack of knowledge about risks or need for further information for safe and effective medicines use (*N* = 69, 26.7 %). The majority of DRPs could be addressed by sole patient counseling (58.9 %). Some pharmacists, however, also intervened by directly changing a patient’s care plan in order to optimise the administration of a therapy (15.5 %), adjust the dosage or substitute a therapy (3.9 %) (Table 3). Study pharmacists noted at T-0, that 69 patients in the intervention group (31.5 %) already used a weekly dosing aid (WDA) in their daily medicines management and they recommended the implementation of a WDA for three patients (1.4 %) (Table 4). During the first telephone interview at T-2, 198 patients stated to own a WDA (47.6 %); 173 of them regularly used the aid (41.6 %), while seventeen patients mentioned a sometime use, e.g. during holidays (4.1 %); eight patients did not use the WDA at all (1.9 %). Until the end of the PMC study (T-28), one patient in the intervention group (0.6 %) and four patients of the control group (2.1 %) newly received a WDA as a result of the PMC. When asked at T-2, 74 (42.8 %) of 173 patients, who had originally been recommended to use a dosing aid, reported that the pharmacy initiated the use of a WDA. Another 54 (31.2 %) bought the WDA themselves, while 20 (11.6 %) received the aid from a hospital. Out of these 173 WDA used at T-2, 158 were independently managed by the patient himself (91.4 %), get refilled by their partner (*N* = 13, 7.5 %) or another third party (*N* = 2; 1.1 %). Thereby, men (*N* = 77) were significantly more often supported by their partners (*N* = 12) than vice versa (N_Women_ = 94, N_Support_ = 1; *p* < 0.001).

**Table 3:** Drug-related problems addressed during PMC at T-0 in intervention group (*N* = 258)

	N	%
Drug-related problems		
Potential	149	58
Manifest	109	42
Urgency rated by the study pharmacist		
High	36	14
Medium	113	43
Low	109	43
Recommendation accepted by patient		
Yes	219	85
No	25	10
Unclear	14	5
Causes of pharmacists’ interventions		
Insufficient adherence	69	26.7
Patient needs information about safe and effective use of his medicines	50	19.4
Patient needs information about potential medicines’ adverse drug reaction	19	7.4
Inappropriate timing or frequency of administration	18	7.0
Under-dosed therapy	15	5.8
Drug-drug/drug-food interaction	14	5.4
Adverse effect	12	4.7
Inappropriate therapy duration	10	3.9
Inappropriate drug administration	9	3.5
Patient needs information about lifestyle, nutrition or empowerment	8	3.1
Not received treatment	7	2.7
More cost-effective therapy available	5	1.9
No concordance with guidelines or contraindication	4	1.6
No dose adjustment because of pathological changes (renal/liver failure)	4	1.6
Not indicated drug or duplication	3	1.2
Incomplete patient documentation	3	1.2
Over-dosed therapy	3	1.2
Prescribed drug not available	2	0.8
Inappropriate monitoring	1	0.4
Not classifiable	2	0.8
Description of pharmacist's interventions		
Counseling of patient, training	152	58.9
Optimisation of administration	40	15.5
Information to other caregivers	24	9.3
Dose adjustment	12	4.7
Substitution of a therapy	10	3.9
Therapy started/restarted	7	2.7
Therapy stopped	7	2.7
Therapy monitoring	3	1.2
Clarification in the patient history	2	0.8
Not classifiable	1	0.4

**Table 4:** Overview of weekly dosing aids in use during study

	Intervention		Control		*p*Value
T-0 (assessed through pharmacist during PMC)	72^a^	*N* = 218	-		-
T-2 (assessed through telephone interview)	83	*N* = 202	90	*N* = 214	0.838
T-16 (assessed through telephone interview)	90	*N* = 198	98	*N* = 203	0.699

^a^From which three were newly implemented through PMC

### Objective adherence

**Fig. 4:**
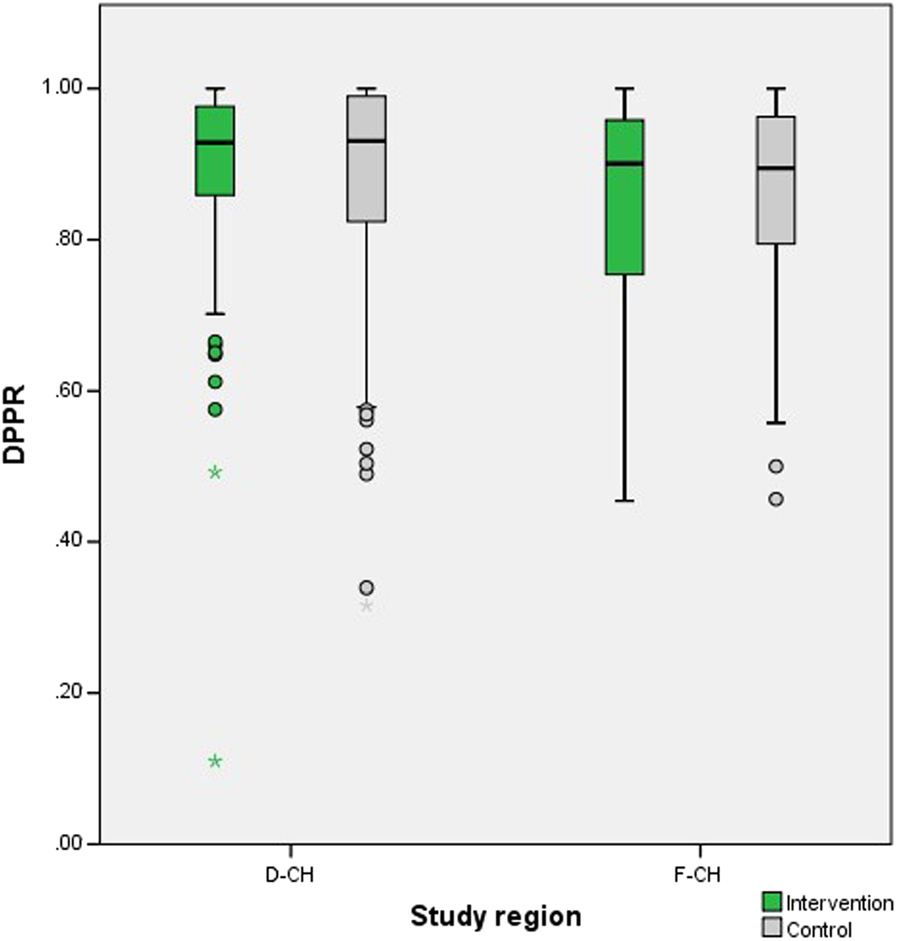
Box plot of DPPR of patients stratified by the German (D-CH, *N* = 199) and the French part of Switzerland (F-CH, *N* = 94)

Out of 2’453 chronic therapies registered in the PMC protocol at T-28, 1’020 (41.6 %) met inclusion criteria for the calculation of their Medication Possession Ratio (MPR) using the defined algorithm (Additional file 2). Sub-analysis of therapies inert to dose adjustments or splitting (and therefore with highest expected validity for calculation of MPR) showed consistent, but no significant trend for improved adherence rates in the intervention group (Table 5). For 212 out of 1'020 therapies (20.8 %) the MPR was < 80 % (intervention *N* = 96 (19.5 %) and control group *N* = 116 (22.0 %), *p* = 0.318). Out of all therapies, the Daily Polypharmacy Possession Ratio (DPPR) was calculated for each individual patient as shown in Table 6 (Additional file 3). Mean DPPR over the whole eligible study population was 87.3 (*N* = 293, SD = 14.250). In both, intervention and control group, the DPPR in D-CH (mean = 88.38, SD = 14.270) was significantly higher compared to that of F-CH (mean = 84.86, SD = 13.972) (*p* = 0.01). Both regions showed no significant improvement of DPPR through the intervention (Fig. 4).

**Table 5:** Objective adherence represented as MPR

	Intervention			Control			
	Mean %	SD	N	Mean %	SD	N	*p*Value
All therapies	88.3	19.03	493	87.5	20.75	527	0.811
Antiplatelets (B01AC)	91.3	16.24	61	85.4	23.75	64	0.119
Proton pump inhibitors (A02BC)	91.8	13.36	43	87.7	18.27	33	0.493

**Table 6:** Objective adherence to polypharmacy represented as DPPR over all patients (*N* = 293)

	Intervention (*N* = 146)		Control (*N* = 147)		
	Mean	SD	Mean	SD	*p*Value
DPPR (%)	88.0	13.31	87.5	20.75	0.906
Number of medicines eligible for DPPR calculation per patient	3.4	1.68	3.6	1.86	0.425

### Subjective adherence

In addition to objective adherence, we asked participants how they estimated their overall adherence on a scale from 0 to 100 %. The mean absolute change of subjective adherence between T-0 and T-2 was +1.03 % in the intervention and −0.41 % in the control group (*p* = 0.058) (Table 7). Sub-analysis revealed, that the number of patients reporting a change of their adherence of more than ±5 points on a scale 0-100 % between T-0 and T-2 was significantly higher in the intervention group (N_Improvement_ = 30; N_Worsening_ = 14) compared to the control group (N_Improvement_ = 20; N_Worsening_ = 24) (*p* = 0.028). Table 8 summarises patient self-report of adherence using validated questionnaires. Between the two telephone interviews T-2 and T-16, mean difference between patients’ beliefs and concerns about their medicines did not change significantly (Intervention = −0.01 (SD 6.609); Control = +0.64, (SD 6.289), *p* = 0.697). At T-16, in total 74 patients had a MMAS-8D score <6 representing low adherence (intervention *N* = 37 (18.9 %), control *N* = 37 (18.3 %)). Moderate adherence (Scores 6–8) was shown in the intervention group for 83 patients (20.8 %) and in the control group for 89 cases (22.3 %). High adherence was present in 154 patients, 78 from the intervention (39.4 %) and 76 from the control group (37.6 %). No significant difference in adherence between the two groups could be observed (*p* = 0.817).

**Table 7:** Subjective rating of adherence during the preceding two weeks

	Intervention			Control			
	Mean %	SD	N	Mean %	SD	N	*p*Value
Patient questionnaire T-0	96.2	8.62	211	96.8	7.05	232	0.204
Telephone interview T-2	97.2	9.31	202	96.4	10.24	213	0.118
Telephone interview T-16	98.5	5.56	198	97.8	7.64	202	0.400
Patient questionnaire T-28	95.5	10.28	178	96.3	9.51	186	0.338

**Table 8:** Summed scores of validated adherence questionnaires at T-2 and T-16

	Intervention			Control			
	Mean	SD	N	Mean	SD	N	*p*Value
T-2							
BMQ Beliefs	20.58	4.463	171	20.99	4.301	181	0.328
BMQ Concerns	9.95	4.249	171	10.30	4.949	181	0.726
Difference Beliefs - Concerns	10.64	5.554	171	10.69	6.494	181	0.612
T-16							
BMQ Beliefs	20.66	4.630	188	21.23	3.958	183	0.369
BMQ Concerns	9.89	5.020	188	9.72	4.583	183	0.872
Difference Beliefs - Concerns	10.77	6.360	188	11.51	5.705	183	0.337
MMAS-8D Score^a^	6.85	1.226	198	6.82	1.237	202	0.817

^a^MMAS-8D Score: 8 = high adherence, 6–7.75 medium adherence, <6 low adherence

### Use of health care resources by patients and unplanned visits at a general practitioner or hospital

According to the notations in the CRF in 18 cases (8.3 %) out of the 258 DRPs addressed at T-0, the study pharmacist contacted the responsible general practitioner (*N* = 17) or an indicated specialist (*N* = 1) to discuss or inform about issues revealed through the PMC. A phone call was reported in six cases (33.3 %), the other issues were addressed by Fax (*N* = 5, 27.8 %), Email (*N* = 1, 5.6 %), referral letter (*N* = 1, 5.6), otherwise (*N* = 3, 16.7 %), not specified (*N* = 2, 11.1 %). Four out of 18 physicians did not respond to the pharmacists’ initiative (22.2 %). The remaining 14 (77.8 %) gave feedback on the addressed issues. Nine fully accepted the pharmacists’ recommendations (64.3 %), one partially (7.1 %), and two rejected the recommended intervention (14.3 %). In two cases, the implementation of the recommendations remained unclear (14.3 %). During the study period, patients reported a total of 209 unplanned visits at a general physician or hospital, showing no significant difference between study groups (Table 9). The same was observed for the incidence of falls during the study.

**Table 9:** Patient reported unplanned visits at general practitioner or hospital and falls during study period

	Intervention		Control		
Unplanned visits …	N_YES_	N_Total_	N_YES_	N_Total_	*p*Value
… from T-0 - > T-2	14	202	10	214	0.324
… from T-2 - > T-16	50	198	44	203	0.398
… from T-16 - > T-28	46	181	45	191	0.678
Incidence of at least one fall until T-28	31	17.7 %	30	15.9 %	0.638
… thereby injured	17	54.8 %	15	50.0 %	0.705

## Discussion

Our study presents initial findings on a newly implemented pharmacist-led medication review service, called Polymedication Check (PMC) with respect to impact on patients’ adherence. The multicentre parallel group randomised controlled trial was conducted in community pharmacies with very low to moderate experiences in providing medication reviews. This paper presents results from multiple in-depth assessments focusing on patients’ adherence and drug-related problems; humanistic outcomes and the patients’ as well as pharmacists’ perspectives will be dealt with in a second publication.

### Study population

Recruitment of study pharmacists posed no problem; all recruited pharmacists attended the required training session. However, experience with providing a PMC proved to be unequal with only 28 % of pharmacists featuring prior experience in conducting >5 PMC and even 34 % with no prior experience at all. Nevertheless, a majority of the study pharmacists who finally started to enrol patients in the project was highly motivated to participate in this evaluation study despite the complexity of the study protocol and their lack of experience with participation in randomised controlled trials. In all regions a suitable sample of pharmacies was involved into the study. The demographics and characteristics of the participating pharmacies were in line with the total of Swiss pharmacies regarding organisational form of ownership and gender compared to RoKA report 2012 [49] (personal ownership and group (study: 74.1 % vs. RoKA: 69.6 %) or chain (25.9 % vs. 30.4 %), women (75.0 % vs. 80.0 %)). The estimated number of ten patients recruited by each study pharmacist was not reached by most pharmacists (Median 7; Range 1–17) despite up to six months of recruitment period per study region. During the study, six study pharmacies cancelled participation before they started recruiting patients and five more dropped out during the study; as a consequence follow-up of their patients was impossible. Patient dropouts were fairly low (17.3 %), evenly distributed across both study groups and caused by an expected pattern of comprehensible reasons, so there is little concern for a selection bias due to selective dropouts. The reported causes were both rare and typical, such as patients’ moving away or being unable to continue due to health reasons.

### Impact of the Polymedication Check

The primary outcome objective adherence showed no significant improvement in the PMC group (mean MPR 88.3 % vs 87.5 % in the control group (*p* = 0.811)).

The adherence in the control population was already at an unexpectedly high rate of 87.5 %, leaving only little room for improvement in the intervention group. This made it nearly impossible to observe the 5 % increase in objective adherence, on which the power calculation was based. Notably, in the intervention group a higher percentage of patients showed more than 5 % increase of subjective adherence compared to the controls. This effect only appeared shortly after the intervention and could not be observed again in the further course of the study.

Our results show that during the PMC non-adherence to medication was the most frequent issue addressed in 26.7 % of PMC cases, followed by a need for information about safe and effective medicines use (19.4 %) or improvement of awareness for risks and adverse effects of therapies (7.4 %). Previous research has shown that adherence counseling was included in only 6.7 % of the reported cases of unspecific pharmacist-patient contacts in usual care [14]. This pattern of detected and discussed drug-related provides an important indication on the impact of the PMC and proves appropriateness of the concept of the PMC regarding its aim at triggering adherence and knowledge issues as topics for individual counseling. The filling of a patient’s medicines into a WDA could be implemented in only very few patients (1.4 %). This unexpected result can be explained with a) the pharmacist judged the patient sufficiently well-organised without a WDA, b) the patient already used a WDA in self-management (which was the case in 42 % of patients in our study) or c) the patients were not willing to delegate the preparation of their medicines to the pharmacist. There is a necessity for guiding a comprehensive assessment of patient needs (self-management of a WDS versus WDA provided by the pharmacy) and differentiating between the active recommendation by the pharmacists and the refusal by the patient. The implementation rate of WDA in patients with chronic polypharmacy revealed in our study, still offers room for improvement; recent surveys in Canada could show that 75 % of patients in a comparable community sample stated to regularly use a WDA [50]. With respect to the interface between pharmacy and GP, 18 out of the 258 cases of detected and addressed DRPs in the PMC group at T-0, cases triggered a consultation with the patient’s GP (7.0 %), leading in 77.8 % to an interprofessional collaboration and discussion of patients’ DRP with high acceptance rate of pharmacists’ recommendations (71.4 %). Still, considering the recommendations without feedback or acceptance by the GP (*N* = 8), the overall implementation rate of 44.4 % is comparable to a study of Kempen et al. [51], who reported implementation rates of 42 %. Such low implementation of recommendations will decrease efficacy of any intervention substantially. However, it can be deduced that the pharmacists were able to solve more than 90 % of the patients’ issues independently.

Unlike reported in previous studies [52], no harmful effect of the PMC intervention as reflected by the non-significant group differences in unplanned hospital admissions or in visits to the GP (Table 9) could be observed. This observation is meaningful when looking at the frequency of contacts of pharmacists with the prescriber resulting from a PMC (8.3 %) and considering that only a few of the pharmacists’ recommendations (14.3 %) were rejected. A significant number of DRPs were discovered and solved through study pharmacists providing a PMC (Table 3).

### Reasons why we did not detect a significant effect

Overall, the study remained underpowered: The initial estimation of the impact on adherence of the PMC was set on 5 % with a baseline at around 60 %. This assumption was based on the results of other studies from different countries and settings [47]. The unexpected high adherence observed in the control group allowed only little improvement. Thus, a sole increase of the study population, e.g. through an extension of the recruitment period, would remain ineffective. A more effective and internationally accepted approach to enhance the efficacy of medication reviews would be the targeting of patients at risk [9]. The high rate of implemented WDA at T-2 (42 %) (Table 4) indicates an already improved patient’s self-management mostly initiated through pharmacists before study start. It was a deliberate decision not to focus on patients with specific diseases or drugs in this first evaluation since the service might be offered to every patient meeting the inclusion criteria for a PMC. Since the inclusion criteria were non-specific in terms of risks for non-adherence, it must be assumed, that already well-organised patients with established therapies were included in this study. Further on, patients with the highest need for intervention with manifest non-adherence might not have been motivated to be part of a clinical trial that explicitly aimed at uncovering individual weaknesses in the correct administration of medicines. Experiences with the MUR service from UK resulted in the development of specific interventions for various patient populations, offering to the health care provider a structured and focused flow chart supporting the process of screening for pharmaceutical care issues [53]. Thus, applying more specific criteria in addressing the medication reviews to patients with higher risk for drug-related problems would probably increase the impact of the intervention.

Medication reviews such as the PMC are a screening method aiming at detecting drug-related problems, and the corresponding interventions are unspecific. Thus, in a first step, this service only results in a number of drug-related problems detected or number of referrals etc. Looking at clinical outcomes, only well planned and monitored interventions can have an impact. The current PMC protocol specifies the provision of a weekly dosing system filled by the pharmacy as its main intervention. This intervention, though known to be effective [54], was offered only to very few patients (1.4 %). All other interventions such as delivery of a medication plan or check of correct use of an asthma device are not foreseen in the protocol and hence could not been evaluated. On the other hand, explicit listing of such predefined interventions on the protocol would probably trigger more frequent provision of such services. So far, the intervention part is insufficiently specified in the current guideline, and especially not well supported by the current PMC protocol.

### Strengths

First, the randomised controlled trial design is a distinct strength of this study. Second, the trial was performed under real-life conditions with a representative sample of pharmacists from different regions, including the French-speaking part of Switzerland with differences related to health care (i.e. density of pharmacies, preferred way of medication supply), cultural and socioeconomic factors. Thus, the results of the present study are likely to be highly generalisable. Third, patients’ adherence was measured using several validated instruments providing internal validity. Fourth, the in-depth telephone interviews on patient’s acceptance and knowledge were performed by trained independent clinical psychologists, blinded to the intervention. Fifth, patients’ written self-reports were blinded to the pharmacists; thus a Pygmalion effect could be excluded.

### Limitations

First, due to restricted financial means, the study period to investigate the objective adherence was limited to just 28 weeks. With regard to the common package size of 100 tablets for long-term medication, the short study duration offered only two refills to be considered for evaluation of adherence. Newer guidelines suggest follow-up periods of 1–2 years or more to capture long term non-adherence [55]. Second, patients enrolled in clinical trials may be more conscientious than the average patient. During the consent process, patients were told that the purpose of the study was to learn more about their daily medicines use and that their adherence to medication was observed. Thus, all our patients knew they were being monitored, which on the one hand may have led to a higher baseline in self-reported adherence at study start and also during follow-up in both groups of our study patients compared to other patients. The pharmacists on the other hand knew that they were being studied, which may have led them to increase their efforts in delivering pharmaceutical care, notably for both groups. This is known as the Hawthorne effect: a psychological response in which subjects in a research study change their behaviour simply because they are subjects in a study, not because of the research treatment [56]. Thus, the heightened awareness of the patients and also of the pharmacists about the study setting could have influenced the medicines intake for the prospective time. Such influence can only be eliminated through a randomisation at the level of the pharmacy – a procedure posing other problems of bias as well. In order to avoid selection bias by the pharmacists, patients were selected at random solely fulfilling the PMC-criteria and not because of an increased risk or any indicators for manifest non-adherence. Third, because of time constraints and limited resources the recruitment was stopped before the intended number of patients was recruited.

### Implications for practice

In line with other authors [9, 55], we recommend to ensure efficiency and efficacy to reconsider and adapt the service on various levels: First, the service should be more tailored to patients at higher risk for drug-related problems, such as patients with respiratory diseases, diagnosed cardiovascular disease, regularly being prescribed at least four medicines etc. In addition, focusing on patients recently discharged from hospital, or who had changes in their medicines regimen would provide more opportunities to screen for manifest DRPs possibly before the start of a risky treatment. Ideally, these patients would receive a medication review within a very short time (e.g. a few days) after the start and are followed by a follow-up meeting (face-to-face or by telephone call) to check for handling issues and implementations of the recommendations. Second, after detecting the patient at risk for clinical relevant drug-related problems, we recommend to proceed with validated, structured and standardised interventions. This process should allow a follow-up to ensure implementation of pharmacists recommendations (according to the pharmaceutical care process, see also the New Medicines Service from the NHS, UK) [57]. The PMC protocol form should include the documentation of the recommendations or follow-up interventions in a more specific structure. Thus, the current process of the PMC as a service and its protocol need to be re-engineered. Third, pharmacists had no training and supervision when providing the service. An implementation program focusing on the main barriers of the service could still encourage pharmacists to provide PMC in the future. A responsible professional body for coaching and answering frequently asked questions is needed. Qualification and/or accreditation of involved health care providers might be considered to ensure high quality and safe interventions on patient level. Continuing education should be strengthened through systematic integration of PMC cases into practice-oriented teaching. For distinct problems or care issues structured guidance should be developed.

## Conclusion

For the first time in the Swiss health care system, a newly implemented cognitive service of community pharmacists underwent an in-depth evaluation process in daily life. The service showed no significant improvement on objective adherence in the observed population. Reasons for not being able to demonstrate significant positive effects are likely to depend on a) an unintentional selection of patients with very high adherence and low risk for drug-related problems causing insufficient power and b) on a low level of experience with providing the PMC among the recruited pharmacists.

However, based on the study results, we conclude that the so called Polymedication Check as a pharmacist-led medication review i) was able to address a significant number of drug-related problems concerning adherence issues and need for knowledge improvement and ii) showed no further financial burden to the Swiss health care system as there was no harm induced and pharmacists’ interventions did not cause additional consultations with other healthcare professionals. Re-engineering of the service should focus on the inclusion criteria to target the patients with highest risk for non-adherence and on the improvement of pharmacists’ skills in implementing weekly dosing aids.

### Availability of data and materials statement

The datasets concerning the primary outcome and thereby supporting the conclusions are included within the article and its additional files.

## Additional files

Additional file 1: Swiss Polymedication Check. (PDF 72 kb) Additional file 2: Data Medication Possession Ratio (MPR). (XLSX 42 kb) Additional file 3: Data Daily Polypharmacy Possession Ratio (DPPR). (XLSX 18 kb)
