# Supplementary material for: Impact of a community pharmacist-led medication review on medicines use in patients on polypharmacy - a prospective randomised controlled trial
Source: BMC Health Serv Res. 2016 Apr 23;16:145. doi: 10.1186/s12913-016-1384-8 (PMC4842295; doi:10.1186/s12913-016-1384-8)
Supplement: Additional file 1: — Swiss Polymedication Check. (PDF 72 kb) [file 12913_2016_1384_MOESM1_ESM.pdf]

Swiss Polymedication Check

Evaluation study ‘evalPMC’ [NCT01739816]

Pat.-ID:	Date of birth: _____ / _____ / _________	Gender: ☐ male ☐ female
The patient currently takes four or more prescribed drugs a day during a period for at least three months ☐		

1. Check Start time: _____._____ h

	Current prescribed drugs [this check is based on patient's information and or sources of the pharmacy's database]	Assessing the need for counseling concerning medicines use: Know **how to dose** [interval]	Know **why**	Know **how long**	"Do you sometimes **forget to take** this medicine?"	**Counseling** through pharmacist needed?	**Comments** [continue on the back if needed]
1	Name, dosing, and galenic formulation New? ☐	__-__-__-__	Yes☐ No☐	☐ On demand ☐ Temporary use ☐ Long-term use	Yes☐ No☐	Yes☐ No☐	
2	Name, dosing, and galenic formulation New? ☐	__-__-__-__	Yes☐ No☐	☐ On demand ☐ Temporary use ☐ Long-term use	Yes☐ No☐	Yes☐ No☐	
3	Name, dosing, and galenic formulation New? ☐	__-__-__-__	Yes☐ No☐	☐ On demand ☐ Temporary use ☐ Long-term use	Yes☐ No☐	Yes☐ No☐	
4	Name, dosing, and galenic formulation New? ☐	__-__-__-__	Yes☐ No☐	☐ On demand ☐ Temporary use ☐ Long-term use	Yes☐ No☐	Yes☐ No☐	
5	Name, dosing, and galenic formulation New? ☐	__-__-__-__	Yes☐ No☐	☐ On demand ☐ Temporary use ☐ Long-term use	Yes☐ No☐	Yes☐ No☐	
6	Name, dosing, and galenic formulation New? ☐	__-__-__-__	Yes☐ No☐	☐ On demand ☐ Temporary use ☐ Long-term use	Yes☐ No☐	Yes☐ No☐	
7	Name, dosing, and galenic formulation New? ☐	__-__-__-__	Yes☐ No☐	☐ On demand ☐ Temporary use ☐ Long-term use	Yes☐ No☐	Yes☐ No☐	
8	Name, dosing, and galenic formulation New? ☐	__-__-__-__	Yes☐ No☐	☐ On demand ☐ Temporary use ☐ Long-term use	Yes☐ No☐	Yes☐ No☐	
9	Name, dosing, and galenic formulation New? ☐	__-__-__-__	Yes☐ No☐	☐ On demand ☐ Temporary use ☐ Long-term use	Yes☐ No☐	Yes☐ No☐	
10	Name, dosing, and galenic formulation New? ☐	__-__-__-__	Yes☐ No☐	☐ On demand ☐ Temporary use ☐ Long-term use	Yes☐ No☐	Yes☐ No☐	

**Self-medication** Yes ☐ No ☐							
A	Name, dosing, and galenic formulation New? ☐	__-__-__-__	Yes☐ No☐	☐ On demand ☐ Temporary use ☐ Long-term use	Yes☐ No☐	Yes☐ No☐	
B	Name, dosing, and galenic formulation New? ☐	__-__-__-__	Yes☐ No☐	☐ On demand ☐ Temporary use ☐ Long-term use	Yes☐ No☐	Yes☐ No☐	
C	Name, dosing, and galenic formulation New? ☐	__-__-__-__	Yes☐ No☐	☐ On demand ☐ Temporary use ☐ Long-term use	Yes☐ No☐	Yes☐ No☐	

2. Counseling / Recommendations

**3. Medication management**	Patient agrees	**4. Interdisciplinary collaboration**		Patient agrees
☐ The patient qualifies for a weekly dosing aid (WDA).<br>He uses ☐ already ☐ newly (since PMC) a WDA. Its refill occurs ☐ through the patient ☐ through the pharmacy.	Yes☐ No☐	☐ **Information** to<br>☐ **Consultation** with<br>☐ **Referral of the patient** to the	physician	Yes☐ No☐
☐ Patient has no need / does not qualify for a WDA.	Yes☐ No☐	☐ **Other interventions after PMC**<br>[...]		Yes☐ No☐

Date: ____ / ____ / ____ End time:____.____ h	Stamp pharmacy / Signature pharmacist:
Patient' signature: ______________________________	

© Pharmaceutical Care Research Group, University of Basel, Switzerland
